# Early-stage triple negative breast cancer: the therapeutic role of immunotherapy and the prognostic value of pathological complete response

**DOI:** 10.37349/etat.2024.00215

**Published:** 2024-02-28

**Authors:** Pierluigi De Santis, Martina Perrone, Chiara Guarini, Anna Natalizia Santoro, Carmelo Laface, Daniela Carrozzo, Gaia Rachele Oliva, Palma Fedele

**Affiliations:** Institute of Experimental Endocrinology and Oncology “G. Salvatore”-National Research Council (IEOS-CNR), Italy; Institute of Experimental Endocrinology and Oncology “G. Salvatore”-National Research Council (IEOS-CNR), Italy; ^1^Oncology Unit, Francavilla Fontana Ceglie Messapica Hospital District, 72021 Francavilla Fontana, Italy; ^2^Department of Medicine and Translational Surgery, Università Cattolica del Sacro Cuore, 00168 Roma, Italy

**Keywords:** Triple negative breast cancer, immunotherapy, pathological complete response, neoadjuvant combination treatment, adjuvant treatment

## Abstract

Triple negative breast cancer (TNBC) represents an aggressive disease associated with a high risk of recurrence after curative treatment and a poor prognosis in the metastatic setting. Chemotherapy was for years the only treatment available in the early and metastatic setting, due to the lack of actionable targets. Clinical practice has changed following the results obtained with the addition of immunotherapy to standard chemotherapy, the development of novel drugs [i.e. antibody-drug conjugates (ADCs)], and the use of targeted treatments for patients carrying germline pathogenic breast cancer susceptibility genes (*BRCA*) *1* or *BRCA 2* variants. The treatment of early-stage disease has had a shift in clinical practice since July 2021, after the Food and Drug Administration (FDA) approval of pembrolizumab in association with chemotherapy as neoadjuvant treatment for TNBC and as a single agent in the subsequent adjuvant setting. This intensive treatment based on the combination of a poly-chemotherapy and an immune checkpoint inhibitor (ICI) led to the improvement of short- and long-term outcomes, but it has highlighted some new unmet clinical needs in the treatment of early-stage TNBC: the selection of the most effective adjuvant therapy and the integration of pembrolizumab with other therapeutic strategies [capecitabine, poly(ADP-ribose) polymerase (PARP) inhibitors] based on the achievement of pathologic complete response (pCR); the identification of predictive biomarkers to select patients who could most benefit from the addition of ICI, to minimize toxicities and to maximize outcomes; the possibility of de-escalating chemotherapy in favor of immune-combo or novel agents, such as ADCs; the role of immunotherapy in estrogen receptor (ER)-low patients. The advent of immunotherapy not only addresses current challenges in TNBC treatment but also holds the promise of a radical transformation in its therapeutic paradigm, enhancing significantly clinical outcomes and offering new perspectives for patients grappling with this aggressive form of breast cancer.

## Introduction

Triple negative Breast Cancer (TNBC) is a histological subtype of breast cancer (BC) characterized by the immunohistochemical lack of expression (< 1%) of estrogen receptor (ER), progesterone receptor (PgR), and human epidermal growth factor receptor 2 (HER2). It accounts for approximately 10–20% of all BC, affecting mainly young, premenopausal women, and individuals with inherited gene alterations, such as BC susceptibility genes 1/2 (*BRCA 1/2*) mutations [1–3]. It notably presents an aggressive biological behavior with a trend to have a higher grade and an often lymph node involvement at diagnosis, an inclination to metastasize after curative treatment, and a poorer prognosis in metastatic setting when compared with other BC subtypes [4, 5].

For decades, treatment for early TNBC has been based on surgery and subsequent adjuvant chemotherapy (CHT) for the reduction of disease recurrence [6]. Therefore, conventional cytotoxic CHT has represented the backbone of systemic treatment in the early TNBC, including neoadjuvant treatment, which used to reduce tumor size in larger tumors increasing the chances of a breast-conserving surgery [7, 8]. In recent years the development of novel therapeutic approaches has been difficult, due to the heterogeneity of TNBC and lack of therapeutic targets [9, 10]. Nevertheless, immunotherapy and poly(ADP-ribose) polymerase (PARP) inhibitors have shown survival benefits in recent studies.

Specifically, combinations of immune checkpoint inhibitors (ICIs) with CHT or other alternative therapeutic compounds could emerge as a successful therapeutic approach in the management of TNBC patients. Despite the progress in ICIs representing a notable milestone in TNBC treatment, additional investigations are necessary to tackle this issue comprehensively. A profound comprehension of tumor subtypes, alongside tumor microenvironment (TME) and in terms of molecular, genetic, and immune aspects, would amplify the potential for developing targeted immunotherapy to achieve superior therapeutic effectiveness, especially in TNBC [11].

Therefore, in this review, we aimed to investigate the role of immunotherapy in early-stage TNBC, the prognostic value of pathologic complete response (pCR) with its therapeutic implications, and the future perspectives regarding the systemic treatment of early TNBC, including the discovery of new biomarkers.

## The landscape of immunotherapy in TNBC

The immune system plays a crucial role in TNBC compared to the other molecular subtypes of BC. Although originally BC was considered non-immunogenic, TNBC has a high immunogenic potential, making it a promising candidate for immunotherapy, especially with ICIs [12, 13]. TNBC immunogenicity is related to intrinsic tumor cell signatures and tumoral surrounding microenvironment features.

Over the last decades thanks to emerging technologies such as next-generation sequencing (NGS), the knowledge of the molecular and genetic background of TNBC improved, bringing to light its intertumoral and intratumoral heterogeneity.

A first classification divided TNBC into six subtypes: basal-like 1 (BL1), basal-like 2 (BL2), mesenchymal (M), M stem-like (MSL), immunomodulatory (IM), and luminal androgen receptor (LAR) [14].

Subsequently, analyzing RNA and DNA-based profiles of 198 TNBC tumors, a four-type classification of TNBC was shaped: basal-like immunosuppressed (BLIS), basal-like immune-activated (BLIA), M and LAR [15]. This classification was further revised with the identification of four specific TNBC subtypes: BL1, BL2, M, and LAR, omitting IM and MSL because of the dependence of these two subtypes on the TME features [14].

In addition, TNBC could be classified into three microenvironment phenotypes or clusters:

(1). Cluster 1: “immune-desert” with poor immune cell permeation, due to a high presence of *MYC* amplifications and, consequently, a lower recruitment of innate immune cells.
(2). Cluster 2: “innate immune-inactivated” characterized by a hyper-activation of phosphatidylinositide 3-kinase/protein kinase B (PI3K-AKT) pathway in tumor cells, low tumor antigen burden and infiltration of deactivated innate immune cells, fibroblasts, and endothelial cells. Clusters 1 and 2 are therefore referred to as “cold tumors”.
(3). Cluster 3: “immune-inflamed”, the so-called “hot tumor” that represents about 30% of TNBCs and is characterized by an abundant adaptive and innate immune cells infiltration and with a high expression of immune checkpoint molecules [16].

The potential “hot” conversion of “cold” tumors could improve the efficacy of cancer immunotherapy. For example, local IM therapies can express a synergistic effect with immunotherapy by acting on components of the TME and immune system function, such as elevating the expression of tumor antigens and increasing the recruitment of activated immune cells in the TME [17].

TNBC cancer cell immunological features include genomic instability and high tumor mutational burden (TMB), resulting in more somatic mutations and neoantigens [18].

Moreover, approximately 10–20% of TNBC harbor *BRCA 1* or *BRCA 2* germinal mutations, with a consequent hereditary deficit in the DNA repair mechanism and strong genomic instability. Several studies have demonstrated that TNBC-carrying BRCA mutations are more sensitive to DNA-damaging drugs such as anthracyclines, but also platinum agents and PARP inhibitors [19–21]. Sensitivity to these drugs was also observed in tumors with alterations in other genes, sharing BRCA-mutant phenotype in the absence of a *BRCA 1/2* mutation, namely “BRCAness” [22, 23].

Tumors with *BRCA 1/2* mutations or BRCAness TNBC are more immunogenic than TNBC without these genetic alterations [24–26].

Compared to the other BC subtypes the immunogenic TME features in TNBC consist of higher levels of vascular endothelial growth factor (VEGF), that promote tumor cell growth and migration such as mitogen-activated protein kinases (MAPKs), tumor-associated macrophages (TAMs), and tumor-infiltrating lymphocytes (TILs), white blood cells that migrate towards the tumor, leading to an important immunogenic effect and consequently that are involved in killing cancer cells [27–29].

TAMs regulate the interaction between the immune system and cancer cells. CD163+ M2 macrophages, which are associated with tumors characterized by higher proliferation and poorer differentiation [30], are more present in TNBC and basal-like BC [31]. A prosperous infiltration of TILs is found in TNBC tumors and the stroma surrounding them, with a recognized predictive and prognostic role, specifically for CD4+ CD8+ T cells [32]. Several studies have reported better response to neoadjuvant CHT (NACT) [33] and better clinical outcomes in BC with high TIL infiltrate [34–39]. Based on this evidence, the international TILs working group started standardizing the evaluation of BC TILs to use it in clinical practice identifying those patients that may benefit from emerging immunotherapies with ICIs or combination therapies [40].

All these TME elements contribute to TNBC immunogenicity which also appears to be closely related to the concept of TMB, depending on the ineffective DNA repair system with the consequent generation of high rates of neoantigens. The upregulated antigen presentation system leads to an increasing number of innate and adaptative immune cells and many cytokines interplaying with cancer cells. However, the exact relationship between TMB, neoantigens, and immune infiltration is not yet completely understood, and some studies have reported an inverse association between immune cells in TME and the rate of somatic copy number alterations [41, 42].

Moreover, although TMB is comparable across the three clusters of TNBC, the “immune-inflated” phenotype is characterized by a higher degree of immune cells in the TME, but also a high expression of immune checkpoints by cancer cells [16]. The rate of TILs, indeed, has been positively related to programmed death ligand 1 (PD-L1) expression [43]. PD-L1 is an immune checkpoint that mediates local immune escape in many tumors inducing saturation of activated T cells. Even if PD-L1 prognostic role is yet controversial [44], however it results more overexpressed in TNBC compared with other BC and it can predict responsiveness to immunotherapy [16].

Therefore, TNBC represents an aggressive BC subtype, associated with high mutational load, high tumor immunogenicity and TME diversity.

## New paradigms in early TNBC: from CHT to immunotherapy

### CHT in adjuvant treatment for TNBC

In early TNBC patients, CHT represents the mainstay of adjuvant and neoadjuvant treatments. Adjuvant CHT is recommended for tumor sizes greater than 1.0 cm and patients with nodal involvement, regardless of tumor size. Therefore, it can be considered for tumor sizes between 0.6–1.0 cm [45]. A recent large meta-analysis demonstrated that adjuvant CHT with anthracyclines-containing regimens plus taxanes, compared with no CHT, can reduce BC mortality rates by about 40% during the first decade after diagnosis. Moreover, regimens with higher cumulative and dose-dense schedules of anthracycline (with granulocyte colony-stimulating factor support) have shown better survival benefits and more reductions in recurrence [46]. Three-weekly docetaxel and paclitaxel can be considered in adjuvant setting, but weekly paclitaxel, in a subgroup analysis, has shown improved outcomes and is preferred for TNBC [47]. In TNBC in frail patients with a known history of heart disease, to minimize the cardiotoxicity of adjuvant treatments, docetaxel combined with cyclophosphamide (TC) has proven to be a viable alternative to doxorubicin and cyclophosphamide (AC), demonstrating a favorable disease-free survival (DFS) [48]. Therefore, there is a broad spectrum of chemotherapeutic treatments for early TNBC that should be customised according to the patient and expected toxicities.

### The role of platinum in adjuvant setting for TNBC

TNBC patients commonly harbor *BRCA 1/2* or BRCAness mutations with a homologous recombination deficiency (HRD) that makes them particularly susceptible to platinum agents due to their ability to hit cancer cells that have deficient DNA repair mechanisms [49–51]. Several retrospective single-center studies have explored the role of adjuvant platinum combined with standard anthracycline and taxane-based regimens, with controversial results not showing clear clinical benefits [52, 53]. Nevertheless, a recent phase III trials have demonstrated a longer 5-year DFS (86.5% *vs.* 80.3%) with similar results in distant DFS and relapse-free survival (RFS) of platinum-containing adjuvant regimens (paclitaxel-carboplatin) compared to a standard anthracyclines-containing regimen followed by taxane, however with no benefit in overall survival (OS) [54].

Another important factor is platinum resistance. Platinum sensitivity may be affected by changes in the hazard ratio (HR) pathway or, in the case of patients with *BRCA 1/2* mutations, by the secondary appearance of new *BRCA 1 or 2* mutations that make cancer cells less sensitive to platinum [55, 56].

Other mechanisms of resistance to platinum compounds are:

(1). Modification of drug transport within the tumor cell, by determining decreased influx or increased efflux.
(2). Increase of detoxification systems.
(3). Decrease of cell apoptosis [57].

Therefore, the benefit of adjuvant platinum-based regimens remains controversial and needs validation by prospective adjuvant ongoing trials.

### Neoadjuvant treatments for TNBC

#### NACT

Several treatment guidelines recommend NACT as the preferred option for stage II or III TNBC and for stage I with a tumor size greater than 1 cm. It can be considered in stage I TNBC with a tumor size from 0.6 cm to 1 cm and/or in the case of tumors with nodal micrometastases. [6, 45]. There is no significant difference in survival benefits between patients receiving neoadjuvant or adjuvant CHT after surgical resection. However, neoadjuvant treatments can be useful for inoperable tumors rendering them operable and they can also downstage patients with operable BC promoting breast-conservation [58, 59]. The use of neoadjuvant treatments provides important prognostic information based on response to therapy. Achieving a pCR, defined as the lack of cancer cells in tissue samples of breast and axillary lymph nodes, after a neoadjuvant treatment, is associated with favorable disease-free and OS in early TNBC, as demonstrated in Collaborative Trials in Neoadjuvant Breast Cancer (CTNeoBC) pooled analysis. In this study, patients with early BC treated with NACT and followed by surgery who obtained pCR (ypT0 ypN0, ypT0/is ypN0) were associated with improved event-free survival (EFS) and OS, especially in TNBC (HR = 0.24 and HR = 0.16, respectively) [33]. Like adjuvant treatment, traditional NACT is based on anthracyclines and taxanes, and a dose-dense regimen is preferred in neoadjuvant settings based on proven improved DFS and OS in a large meta-analysis [60].

In recent years, the use of platinum-based combination regimens has been the focus of neoadjuvant treatment to increase the rate of pCR in TNBC. Three recent studies demonstrated that combining platinum with taxane and anthracycline led to an improvement in the pCR rate in TNBC, with a similar survival benefit [61–63]. In Brightness Trial patients with II–III stage TNBC were randomly assigned to receive paclitaxel alone, paclitaxel and carboplatin and this combination with a PARP inhibitor, veliparib followed by AC. Although the addition of veliparib and carboplatin was associated with an increase of patients who achieved a pCR compared to paclitaxel alone (53% *vs.* 31%, *P* < 0.0001), but not to paclitaxel and carboplatin, this benefit could be related to the addition of the carboplatin [63]. The initial rationale for using the combination of platinum in NACT was that sporadic TNBC can show BRCAness with a major response to platinum regimens [50, 51]. However, the greatest benefit was seen in patients who were germline BRCA wild type, and only a marginal benefit was observed in the germline BRCA mutant subgroup, as was shown in the recent GeparOLA trial. In this trial, patients were randomized to neoadjuvant therapy with paclitaxel and carboplatin *vs.* neoadjuvant therapy with paclitaxel and olaparib (PARP inhibitor). In both arms, the combination of epirubicin and cyclophosphamide was administered next. This study, although limited by a small number of patients enrolled, showed an advantage for the carboplatin arm in patients without BRCA mutation (germ or somatic) and high HRD. The 4-year invasive DFS (iDFS) rate with olaparib-paclitaxel was 81.2% *vs.* 93.4% with carboplatin-paclitaxel (CP) [HR = 3.03; 95% confidence interval (CI) = 0.67–13.67; log-rank *P* = 0.1290]. The 4-year OS rate was 89.2% with the olaparib combination *vs.* 96.6% with carboplatin (HR = 3.27; 95% CI = 0.39–27.20; log-rank *P* = 0.2444). The trend of the iDFS curves was similar in the two treatment arms and independent of germline or somatic BRCA mutation [64].

Platinum combinations are currently recommended for selected patients with TNBC who require adequate local control before surgical resection [45]. A more recent phase III trial presented at the San Antonio Breast Cancer Symposium evaluating the efficacy and safety of adding carboplatin to standard sequential taxane-anthracycline NACT in patients with TNBC who had no evidence of metastatic disease, has observed improvements in terms of DFS (5-year DFS were 70.6% and 64.5% respectively with a HR = 0.79, 95% CI = 0.61–1.02, *P* = 0.073) and OS (5-year OS were 74.0% and 66.7% respectively with a HR = 0.75, 95% CI = 0.57–0.98, *P* = 0.034) with the addition of carboplatin, but these benefits were limited to patients who were 50 years of age or younger. Therefore, the pCR in the intention-to-treat population was 54.5% in the carboplatin arm and 40.3% in the control arm (*P* < 0.001) [65].

The inclusion of platinum agents as NACT for TNBC remains controversial. Long-term outcomes and new prospective studies are needed to clarify the role of platinum agents in this setting.

#### Neoadjuvant immunotherapy

The success of ICIs in metastatic TNBC led to expand their role in neoadjuvant settings. Pembrolizumab and atezolizumab have shown progression-free survival (PFS) benefits in phase III trials in advanced setting [66, 67]. In contrast to atezolizumab that showed conflicting results [68, 69], pembrolizumab consistently showed OS benefits in advanced TNBC [66, 70]. In early-stage TNBC two studies evaluated atezolizumab in neoadjuvant setting. In the NeoTRIPaPDL1 trial, no improvement in pCR was shown with the addition of atezolizumab to a non-anthracycline-containing CHT regimen [71]. More recent Impassion031 phase III study evaluating the association of atezolizumab to a standard NACT (nab-palclitaxel weekly for 12 weeks followed by 4 cycles of AC), has demonstrated a significant improvement of pCR rates in intention to treat (ITT) population (58% in atezolizumab arm *vs.* 41% in placebo arm, *P* = 0.0044), regardless of PD-L1 status, meeting the primary endpoint of the study [72]. Therefore, in early BC the combination of pembrolizumab with paclitaxel-carboplatin followed by anthracycline increased pCR rate and EFS rate in the KEYNOTE-522 trial, representing a turning point for the role of immunotherapy in neoadjuvant therapy of TNBC and establishing pembrolizumab as a standard treatment during neoadjuvant treatment for stage II and III TNBC. The trial evaluated the combination of pembrolizumab (18 cycles, 200 mg every 3 weeks) combined with four cycles of paclitaxel (weekly or 3-weekly) and carboplatin (3-weekly), followed by 3-weekly AC for 4 cycles, compared to placebo with CHT. Pembrolizumab arm showed a 13.6% improvement in pCR [64.8% (95% CI = 59.9–69.5%) *vs.* 51.2% (95% CI = 44.1–58.3%)] and in EFS rate [84.5% (95% CI = 81.7–86.9%) *vs.* 76.8% (95% CI = 72.2–80.7%)], meeting the primary endpoint of the study, regardless nodal involvement and PD-L1 status. The average duration of follow-up is still immature, but a trend of superiority in terms of OS in the pembrolizumab arm was nevertheless detected [73]. Limits of this study are the lack of biomarkers that predict what patient may benefit from the addition of pembrolizumab and the non-utilization of dose-dense schedule of AC which showed superior OS benefit in the neoadjuvant setting in TNBC [74].

Moreover, the recent GeparNuevo trial showed that durvalumab (1,500 mg every 4 weeks) added to NACT consisting of nab-paclitaxel 125 mg/m^2^ weekly for 12 weeks, followed by epirubicin/cyclophosphamide every 2 weeks, in early TNBC significantly improved iDFS (85.6% with durvalumab *vs.* 77.2% with placebo HR = 0.48, 95% CI = 0.24–0.97, stratified log-rank *P* = 0.036) and OS (95.2% *vs.* 83.5% with a HR = 0.24, 95% CI = 0.08–0.72, *P* = 0.006), despite a modest pCR increase and no adjuvant component of durvalumab [75]. Future studies should aim to define the role of immunotherapy in the treatment of early TNBC, to define the ideal duration of these treatments, and should research new biomarkers to personalize treatments.

## Pathological complete response: prognostic role and therapeutic implications

In clinical practice, the achievement of pCR after neoadjuvant treatment is correlated to the improvement of long-term benefits concerning EFS and OS. Its prognostic value is greatest in aggressive tumor subtypes, like in TNBC (EFS: HR = 0.24; OS: HR = 0.16) [33]. Patients who have residual invasive BC after the receipt of NACT have a high risk of relapse. Patients with TNBC who do not experience pCR have an estimated 5-year EFS of 57% and OS of 47% (compared with 90% EFS and 84% OS, respectively, for patients with early-stage TNBC who demonstrate pCR) [76, 77].

After pre-operative CHT and surgical treatment, patients can receive postoperative radiation therapy (RT). Patients with hormone receptor-positive BC [hormone receptor-positive (HR+) BC] are candidates for adjuvant endocrine treatment. However, until recently, no adjuvant CHT was expected as standard in patients with TNBC. Only follow-up was recommended in those who have pCR or in those with residual invasive BC after the receipt of neoadjuvant regimens [78]. To address the unmet clinical need for optimal adjuvant treatment in the subgroup of patients with TNBC at high risk of recurrence (those who have not achieved the pCR after NACT containing anthracycline, taxane, or both), the Capecitabine for Residual Cancer as Adjuvant Therapy (CREATE-X) was designed. The trial did not include only patients with TNBC but also patients with HR+ HER2 negative BC [79]. The results of this phase III trial showed that the addition of adjuvant capecitabine (1,250 mg per square meter of body-surface area, twice per day, on days 1 to 14, every 3 weeks for six or eight cycles) was safe and effective in prolonging DFS and OS among the ITT population. The study showed a superior DFS in the capecitabine group than in the control group (74.1% *vs.* 67.6% at 5 years; HR = 0.70; *P* = 0.01). Therefore, OS was longer in the experimental group: 89.2% *vs.* 83.6% of the patients were alive at 5 years (HR = 0.59; *P* = 0.01). Thirty percent of the patients had triple negative (TN) disease, and they represent the subgroup with poor prognosis (approximately half the patients with TNBC who had a pCR did not have the recurrence of the disease) [33]. The benefit of capecitabine *vs.* control in DFS and OS was notable among this subgroup of patients (HR = 0.58 and HR = 0.52, respectively) [79].

The reflection in the treatment algorithm due to these results was significant.

Some limits of this study are the exclusion of patients who had reached the pCR, for whom only follow-up was indicated, and the lack of efficacy results selected for residual cancer burden (RCB). The RCB quantifies the extent of residual disease after neoadjuvant treatment at the time of surgery. This score uses the diameter of residual disease, percentage of vital tumor cells, and diameter of the largest involved lymph node to calculate the amount of residual disease. It has been validated with distinct prognostic RCB classes in all BC subtypes, with the most significant discriminatory power in TN and Her-2 positive BC. It is categorized as RCB-0 (equivalent to a pCR), RCB-1, RCB-2, and RCB-3, reflecting increasingly larger residual cancer and respective poor prognoses (in terms of EFS) [80]. Finally, the CREATE-X trial did not examine capecitabine efficacy in patients with germline *BRCA 1* or *BRCA 2* pathogenic variants (less than 15% of those enrolled) [79].

OlympiA is a phase III study designed to investigate how the PARP inhibitor olaparib might improve DFS and OS in patients with resected HR+ BC and TNBC with germline *BRCA 1* or *BRCA 2* mutation. It enrolled patients treated with CHT (containing anthracyclines, taxanes or the combination of both) in neoadjuvant or adjuvant setting and randomized them to receive olaparib (orally administered at the dose of 300 mg twice daily) *vs.* placebo for 1 year after surgical resection (and radiotherapy when indicated). Also in this trial, patients with TNBC who underwent NACT followed by surgery were required to have residual invasive BC in the breast and/or resected lymph nodes (non-pCR) [81]. Postneoadjuvant capecitabine was not foreseen in this trial. iDFS, the primary endpoint of the study, was significantly longer among patients assigned to receive olaparib than among those assigned placebo (HR = 0.58; *P* < 0.001). The percentage of patients alive and free of invasive disease at 3 years was 85.9% in the olaparib group and 77.1% in the placebo group. The benefit of adjuvant olaparib was observed irrespective of the germline *BRCA* mutation (BRCA 1 *vs.* BRCA 2), the hormone-receptor status, or the timing of previous CHT (neoadjuvant *vs.* adjuvant) [81] 4-year iDFS for the olaparib group was 82.7% (*vs.* 75.4% in placebo group) and 4-year distant DFS (DDFS) was 86.5% (*vs.* 79.1%). Adjuvant olaparib improves OS, with an HR of 0.68 and a *P* value of 0.009 at 3.5 years of median follow-up, meeting the significance threshold for OS at the second planned interim analysis. The OS benefit at 4 years in the olaparib arm compared with the placebo arm was reported (89.8% *vs.* 86.4%, respectively) [82].

Both studies have defined the standard of adjuvant therapy post-NACT for patients with *BRCA* wild type (CREATE-X) and *BRCA* mutated (OlympiA) TNBC, that did not reach the pCR.

The low percentage of *BRCA* mutated patients enrolled in the CREATE-X, the absence of pre-planned subgroup analyzes for this population do not allow for a description of the efficacy of capecitabine in this subgroup of patients.

Moreover, there are no prospective randomized trials between capecitabine and olaparib to guide the clinical decision in this population, nor combination or sequence data between these two drugs.

It would also be important to consider the potentially severe toxicity profile of such a combination, given their overlapping side effects (in particular, cytopenias).

The treatment paradigm of early TNBC has had a real evolution since July 2021, with the introduction of immunotherapy following the Food and Drug Administration (FDA) approval of pembrolizumab for high-risk TNBC (tumor size > 1 cm but ≤ 2 cm in diameter with nodal involvement or tumor size > 2 cm in diameter regardless of nodal involvement), regardless of tumor PD-L1 expression, in combination with CHT as neoadjuvant treatment, and then continued as a single agent as adjuvant treatment after surgery for a total duration of approximately 1 year [83].

Results from the KEYNOTE-522 study were the basis for this approval, demonstrating a significantly higher rate of pCR at the time of definitive surgery among patients who received pembrolizumab plus NACT than among those who received placebo plus NACT and an improvement in long-term benefits [73, 84]. The aim of the trial was not to identify the contributions of the neoadjuvant and adjuvant treatment phases, so it is difficult to define if these long-term results are related to exposure to adjuvant pembrolizumab or a lesser RCB at the end of the neoadjuvant phase in the pembrolizumab–CHT group.

**Figure 1:**
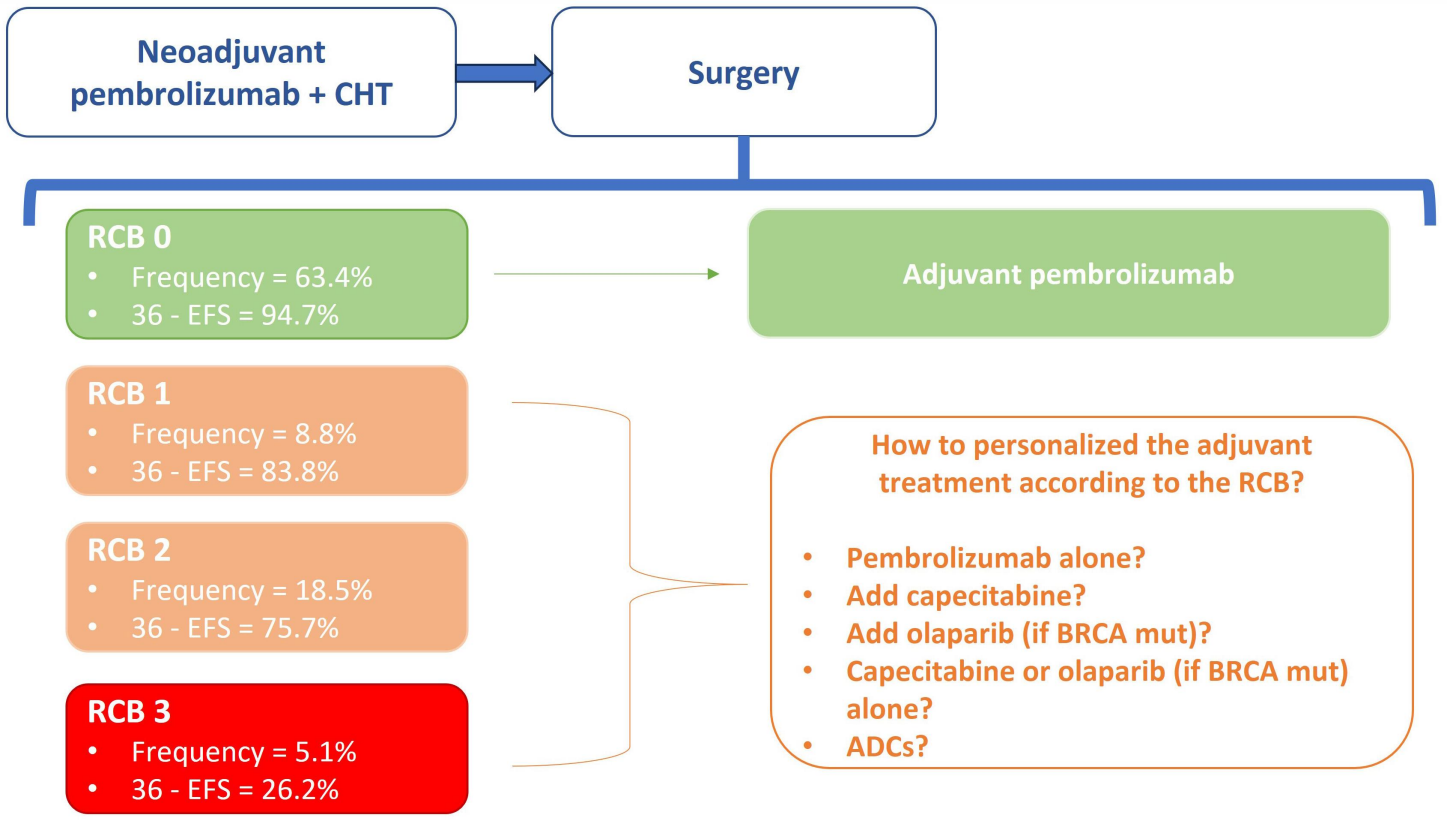
The unmet need for the optimal adjuvant treatment according to RCB [85]

An exploratory analysis of the study then provided data to further describe the prognosis related to the RCB after neoadjuvant experimental treatment (Figure 1) [85].

The HR for recurrence event in subgroups RBO-0, RCB-1, RCB-2, and RCB-3 are respectively 0.70 (rates: 5.2% *vs.* 7.3% in the pembrolizumab + CHT *vs.* placebo + CHT), 0.92 (rates: 17.4% *vs.* 20%), 0.52 (rates: 25.5% *vs.* 44.3%), 1.24 (72.5% *vs.* 69.2%).

The rate of recurrence was numerically lower in all RCB groups with pembrolizumab + CHT, except in the small RCB-3 subset (that is represented by 5% and 7% of the population in the study, respectively in the experimental and control group). Pembrolizumab shifted RCB to lower categories in most patients (RCB-0: 63% *vs.* 56% of patients in the experimental *vs.* the control arm; RCB-1: 9% *vs.* 11%; RCB 18% *vs.* 20%).

No patients in this trial received adjuvant capecitabine, and there are no randomized efficacy and safety data showing that multiagent therapy with pembrolizumab and capecitabine is superior to single-agent therapy in high-risk patients (stage II–III) who did not reach pCR.

At the time, only results from phase II studies in metastatic TNBC demonstrated no new safety signals with this combination [86, 87].

Pembrolizumab has also not been studied in combination with olaparib in the adjuvant setting, for the treatment of patients with *BRCA* mutations. No efficacy data are reported in the literature, even if some safety data are reported in the metastatic setting, in some early-phase studies that have evaluated the combination of PARPis and ICIs, not reporting unexpected toxicities [88, 89].

Prospective trials would be needed to define what is the optimal adjuvant strategy according to RCB (single-agent CHT or poly-CHT), how the clinician should decide between olaparib, immunotherapy or capecitabine in the treatment of the population with *BRCA* mutations and whether these therapies can be administered in combination or sequence, with data in terms of efficacy and safety.

Additional treatment strategies with new drugs are being studied as adjuvant treatment after NACT, with antibody-drug conjugates (ADCs) such as datopotamab deruxtecan (with or without durvalumab in TROPICS-Breast 03, ClinicalTrials.gov identifier: NCT05629585), and patritumab deruxtecan (HER3-DXd) which showed promising clinical response and biological changes in early TNBC [SOLTI TOT-HER3 window of opportunity trial part B, presented at European Society for Medical Oncology (ESMO) Breast 2023], or with ICIs (A-BRAVE trial, NCT02926196 and SWOG S1418/BR006 trial, NCT02954874).

## New biomarkers and frontiers in TNBC

Recent progress in integrating ICIs and novel agents has revolutionized the therapeutic approach for early TNBC. Treatment strategies now emphasize escalating chemotherapeutic agents based on standard neoadjuvant regimens. An example is a phase II trial (ACTRN12617000651381) presented at the San Antonio Breast Cancer Symposium 2022 evaluating in high-risk TNBC, the addition of ipilimumab and nivolumab to neoadjuvant paclitaxel following a suboptimal response to anthracycline-based CHT (< 50% tumor reduction) and resulting in promising objective response rate (ORR) (43.7%) and pCR (18.8%) rates, regardless of PD-L1 status.

However, it is also crucial to identify subgroups of patients with favorable prognoses, where NACT could potentially be de-escalated. Therefore, discovering novel biomarkers to categorize patients with good prognoses and safely de-escalate NACT is essential.

TILs show promise as a biomarker for selecting patients who may have favorable outcomes with treatment de-escalation. In recent trials, higher TILs levels were associated with a higher pCR rate [71, 75, 90] and with a better response [75, 91]. Liquid biopsies, such as circulating tumor DNA (ctDNA), could serve as promising markers for identifying patients who might benefit from de-escalating or escalating neoadjuvant or adjuvant treatment. Rapid ctDNA clearance during NACT in early TNBC is linked to a high likelihood of achieving pCR [92]. Conversely, detecting ctDNA after completing NACT and surgery is associated with higher recurrence rates and poorer prognoses [93]. The use of dynamic biomarkers, such as ctDNA, to guide the choice of treatments in high-risk patients appears increasingly to be an important resource to be exploited in future studies.

Furthermore, ADCs are emerging. Particularly, sacituzumab govitecan (SG) an ADC targeting Trop-2 was approved in metastatic TNBC patients who received ≥ 2 prior systemic therapies in the light of the results of the phase III ASCENT study. In this trial patients were randomized (1:1) to receive sacituzumab govitecan 10 mg/kg via intravenous infusion on day 1 and day 8 of a 21-day treatment cycle or a treatment of physician’s choice (TPC) achieving the primary endpoint (PFS 4.8 *vs.* 1.7 months) and also demonstrating an advantage in terms of OS (11.8 months *vs.* 6.9 months) [94]. Another single-arm phase II trial is evaluating SG and atezolizumab in combination as adjuvant treatment for patients with TNBC who have residual invasive disease after neoadjuvant therapy and detectable ctDNA (ClinicalTrials.gov identifier NCT04434040).

Finally, it is essential to redefine, with new dedicated trials, the role of ER-low (1–9%) BC which, biologically and prognostically very similar to TNBC, could potentially benefit from the addition of immunotherapy to CHT and the role of HER-2 low [score 1+ or 2+ not amplified in fluorescence *in situ* hybridization (FISH)] BC in the light of recent results of efficacy of trastuzumab deruxtecan in advanced BC HER-2 low. Therefore, future studies are likely to expand the armamentarium at our disposal in this setting.

**Figure 2:**
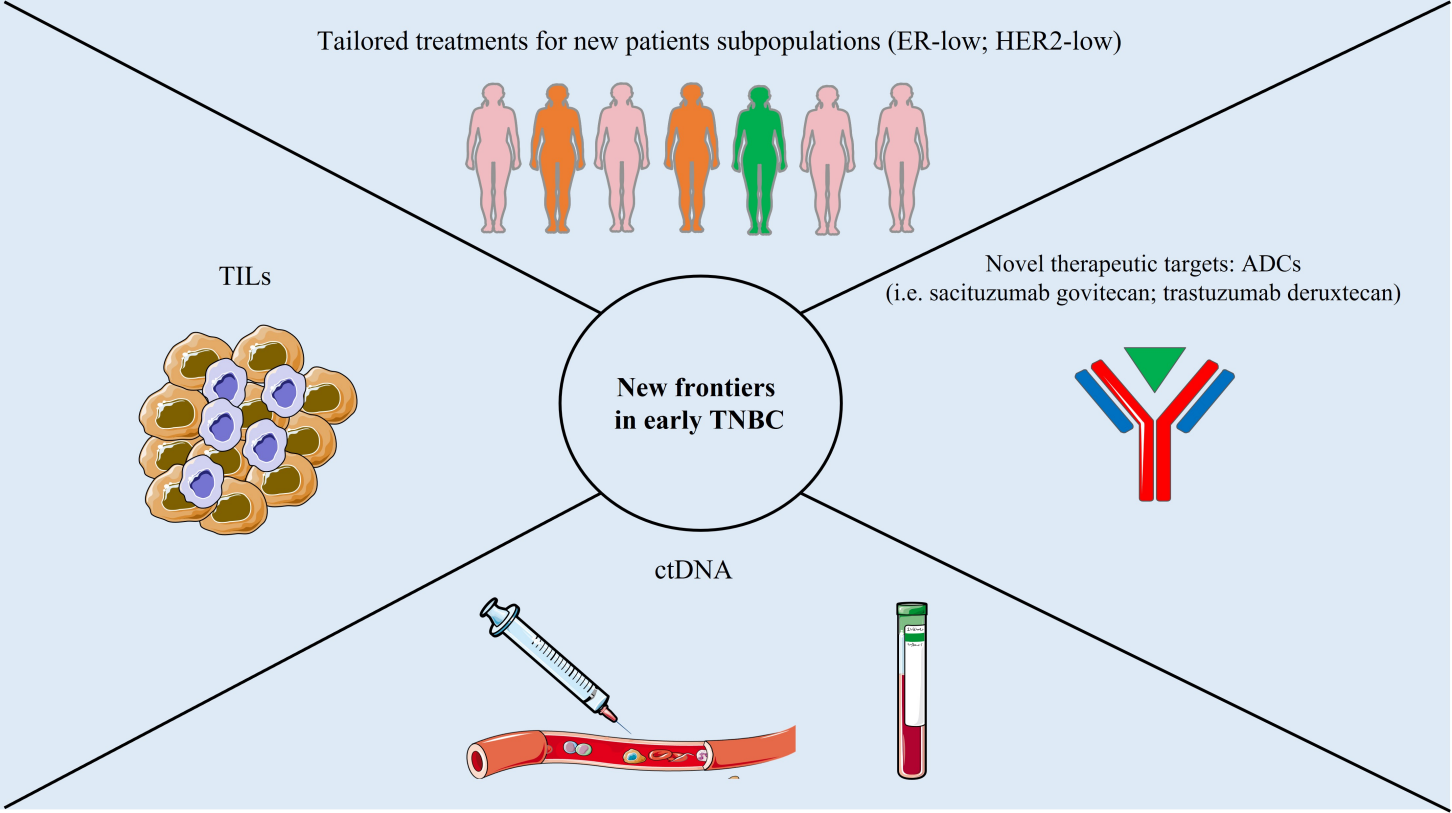
New frontiers in early TNBC

New frontiers in early TNBC are summarized in Figure 2.

## Interpretation and clinical implications

TNBC has long been a challenging disease to treat due to its aggressive behavior and the lack of target therapies [95].

Thanks to recent developments on TNBC, a series of therapeutic targets have been identified for the treatment of metastatic and early setting diseases. Especially for the radically operable disease, the chances of cure are increased with treatments aimed at reducing the odds of recurrence after tumor removal.

Anthracycline and taxane-based poly-CHT remains the standard of treatment, most often administered preoperatively to assess tumor sensitivity. It aims to increase the rate of local control, making it useful to guide breast-conserving surgery and to ensure survival benefits by reaching the pCR.

The introduction of immunotherapy in association with poly-CHT in the neoadjuvant setting has increased the rate of pCR, guaranteeing better results in terms of long-term benefits in the KEYNOTE-522, the pivotal trial that led to the approval in clinical practice of the use of the anti-PD1, pembrolizumab, in the early setting disease (neoadjuvant and adjuvant setting). These clinical findings were based on preclinical investigations that overturned the previous belief that BC was not an immunogenic disease [12].

**Figure 3:**
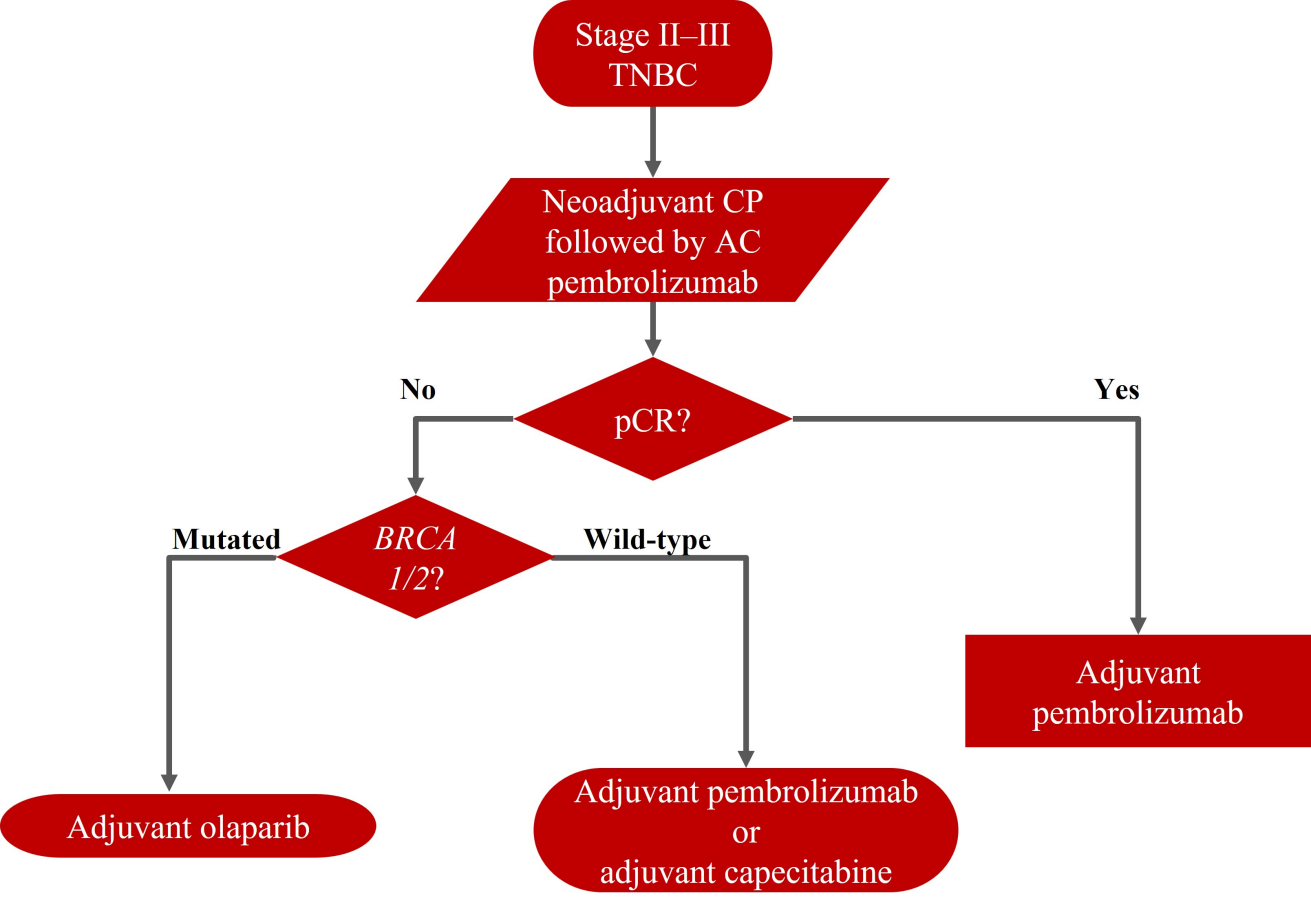
Current treatment algorithm for stage II–III TNBC

The actual need is to define the optimal adjuvant strategy after neoadjuvant chemo-immunotherapy, which must be affected by the patient’s risk of recurrence based on the histological prognostic and evidence after radical surgery, the individual’s tolerance of therapy-induced side effects (Figure 3).

In patients with low RCB and a low overall risk of recurrence, pembrolizumab alone should be continued. In patients with poor prognostic features of high RCB, this strategy may not be the best choice. Patients with high RCB, BRCA wild type, could benefit from capecitabine alone, although it would be reasonable to use a combination of capecitabine and pembrolizumab. Patients with high RCB, germline *BRCA* mutations, could benefit from olaparib (according to the inclusion criteria of the OlympiA trial), although it would be reasonable to use olaparib and pembrolizumab in combination or sequentially.

However, none of these strategies, in monotherapy and/or in combination, have evidence from specific randomized trials after the neoadjuvant immunotherapy. There are no data on efficacy and safety in this setting. Currently, the best schedule is not known, and new data are awaited on new adjuvant strategies.

Extensive efforts will also be required to investigate and expand access to immunotherapy to ER-low populations (ER 1–9%), not included in KEYNOTE-522. It represents a subgroup that does not formally meet the definition of TNBC, but shares biology, with nearly 90% of these tumors harboring a basal-like intrinsic subtype, and prognosis with TNBC and could share the same benefit from the addition of immunotherapy [96, 97].

Furthermore, novel active agents are emerging for the treatment of TNBC and could provide an opportunity for a de-escalation of traditional CHT, the anti-trophoblast cell-surface antigen 2 (Trop2) sacituzumab govitecan that is currently being investigated in the early setting, including in combination with immunotherapy in the ASPRIA trial (ClinicalTrials.gov identifier NCT04434040).

## Conclusions

This review highlights the multitude of advances in the treatment of early-stage TNBC and the important issues raised.

The management of triple-negative breast cancer (TNBC) has seen notable advancements with the identification of therapeutic targets and successful integration of immunotherapy in neoadjuvant treatment. However, the current challenge lies in determining the optimal adjuvant strategy post-chemo-immunotherapy, tailoring decisions to individual patient characteristics and prognostic factors. The uncertainty surrounding the efficacy and safety of these strategies necessitates further randomized studies, while ongoing research explores novel approaches, such as the potential use of innovative agents like sacituzumab govitecan in the context of de-escalating traditional CHT. The imperative to extend access to immunotherapy to subgroups, such as those with low ER expression, holds crucial promise, paving the way for a more personalized and targeted future direction in TNBC treatment.

In the next few years, it will be necessary to design new prospective clinical trials and wait for the results of those in progress, for a better knowledge of the efficacy of combination therapies, therapeutic sequences and new target drugs for the treatment of a disease which up to a few years ago was considered “untargetable”. This should be accompanied by a commitment to biomarker discovery, which could help the oncologist make the best decision for patient care.

## Abbreviations

AC: doxorubicin and cyclophosphamide
ADCs: antibody-drug conjugates
BC: breast cancer
BRCA: breast cancer susceptibility genes
CHT: chemotherapy
CI: confidence interval
CREATE-X: Capecitabine for Residual Cancer as Adjuvant Therapy
ctDNA: circulating tumor DNA
DFS: disease-free survival
EFS: event-free survival
ER: estrogen receptor
FDA: Food and Drug Administration
HER2: human epidermal growth factor receptor 2
HR: hazard ratio
HR+: hormone receptor-positive
ICI: immune checkpoint inhibitor
iDFS: invasive disease-free survival
IM: immunomodulatory
LAR: luminal androgen receptor
M: mesenchymal
NACT: neoadjuvant chemotherapy
OS: overall survival
PARP: Poly(ADP-ribose) polymerase
pCR: pathologic complete response
PD-L1: programmed death ligand 1
RCB: residual cancer burden
TILs: tumor-infiltrating lymphocytes
TMB: tumor mutational burden
TME: tumor microenvironment
TNBC: triple negative breast cancer

## Declarations

### Copyright

© The Author(s) 2024.
